# Study of Interactions Between Gadolinium-Based Contrast Agents and Collagen by Taylor Dispersion Analysis and Frontal Analysis Continuous Capillary Electrophoresis

**DOI:** 10.3390/ph17121633

**Published:** 2024-12-05

**Authors:** Chutintorn Somnin, Joseph Chamieh, Laurent Leclercq, Christelle Medina, Olivier Rousseaux, Hervé Cottet

**Affiliations:** 1 IBMM, University of Montpellier, CNRS, ENSCM, 34095 Montpellier, France; chutintorn.somnin@etu.umontpellier.fr (C.S.); laurent.leclercq@umontpellier.fr (L.L.); 2 GUERBET, Research and Innovation, 16 rue Jean Chaptal, 93600 Aulnay Sous Bois, France; christelle.medina@guerbet.com (C.M.); olivier.rousseaux@guerbet.com (O.R.)

**Keywords:** gadolinium-based contrast agents, interaction, collagen, Taylor dispersion analysis, frontal analysis continuous capillary electrophoresis

## Abstract

Background: Gadolinium-based contrast agents (GBCA) are widely used in magnetic resonance imaging (MRI) to enhance image contrast by interacting with water molecules, thus improving diagnostic capabilities. However, understanding the residual accumulation of GBCA in tissues after administration remains an area of active research. This highlights the need for advanced analytical techniques capable of investigating interactions between GBCAs and biopolymers, such as type I collagen, which are abundant in the body. Objective: This study explores the interactions of neutral and charged GBCAs with type I collagen under physiological pH conditions (pH 7.4) using Taylor dispersion analysis (TDA) and frontal analysis continuous capillary electrophoresis (FACCE). Methods: Collagen from bovine achilles tendon was ground using a vibratory ball mill to achieve a more uniform particle size and increased surface area. Laser granulometry was employed to characterize the size distributions of both raw and ground collagen suspensions in water. TDA was used to assess the hydrodynamic radius (*R_h_*) of the soluble collagen fraction present in the supernatant. Results: From the TDA and FACCE results, it was shown that there were no significant interactions between the tested GBCAs and either the ground collagen or its soluble fraction at pH 7.4. Interestingly, we also observed that collagen interacts with filtration membranes, indicating that careful selection of membrane material, or the absence of filtration in the experimental protocol, is essential in interaction studies involving collagen. Conclusion: These findings bring valuable insights into the behavior of GBCAs in biological systems with potential implications for clinical applications.

## 1. Introduction

Gadolinium-based contrast agents (GBCA) are complexes of Gd^3+^ ions used in magnetic resonance imaging (MRI) to enhance image contrast by interacting with water molecules and decreasing proton relaxation times [1]. The Gd^3+^ ion, which has a large magnetic moment and long relaxation time, is toxic in its free form [2] but becomes safe and effective when chelated with polyaminocarboxylic acid ligands, forming stable complexes. These complexes are hydrophilic, thermodynamically stable, and kinetically inert. GBCA, introduced in the 1980s [3], is essential in diagnosing tissue and vascular abnormalities, especially for brain tumors. Gadolinium-based contrast agents (GBCAs) are classified into linear and macrocyclic compounds, with macrocyclic agents—including polyazamacrocyclic structures—featuring a closed, cage-like molecular structure that provides higher thermodynamic stability and kinetic inertness compared to linear agents. This stability reduces their affinity for endogenous metal ions and minimizes the risk of gadolinium release [4,5,6] with typical stability constants K_Gd_ of 10^15^–10^19^ at pH 7.4 [7]. Despite these structural differences, all GBCAs enhance MRI contrast, though they vary in stability, elimination pathways, and the potential for gadolinium retention in tissues. 

The biological fate of GBCAs involves rapid diffusion after intravenous injection, distribution across anatomical regions, and elimination primarily through the kidneys via glomerular filtration, with a plasma half-life of approximately 1 to 2 h [8,9,10]. However, residual gadolinium persists in humans and animals after GBCA administration, raising concerns since 2014 about its presence in the central nervous system (CNS) and in the skin and bones [11]. A recent report indicates that GBCA also accumulates in the kidneys, an effect not detectable by MRI [12]. To better understand these biological processes, studying the interactions between GBCA and biopolymers present in the body is essential. This requires the development of new analytical methods for the study of both neutral and negatively charged GBCAs. Previous studies have reported interactions between some GBCAs and various biomolecules, such as human serum albumin (HSA) [13,14], lysozyme [15,16,17], and also collagen [18,19].

Collagen, which constitutes approximately 25% of the body’s total protein, is predominantly type I collagen, making up about 80% of the total collagen [20]. The interaction of Gd-DOTA (Dotarem^®^), Gd-BT-DO3A (Gadovist^®^), Gd-HP-DO3A (ProHance^®^), and Gd-HB-DO3A (a research complex not approved for human use) with type I collagen (bovine achilles tendon) was investigated using ultrafiltration and dialysis with cellulose membranes, followed by analysis using micellar electrokinetic capillary chromatography (MEKC) [18]. The study reported similar affinities of the tested GBCAs for collagen, with the following ranking: Gd-DOTA > Gd-BT-DO3A > Gd-HP-DO3A. Gd-HB-DO3A, a slightly more lipophilic analogue of Gd-HP-DO3A, exhibited a noticeable interaction with collagen, though this interaction was significantly weaker compared to other macrocyclic GBCAs [19]. Recently, Ibhagui et al. developed a human collagen-targeted protein MRI contrast agent (hProCA32.collagen) for noninvasive early diagnosis and drug treatment monitoring of pulmonary fibrosis. They reported a binding constant of about 0.18 µM between hProCA32.collagen and collagen type I by ELISA assay [21]. To our knowledge, no other research group has reported affinity constants for interactions between non-targeted GBCAs and collagen.

Taylor dispersion analysis (TDA) method has recently gained attention for determining diffusion coefficients and hydrodynamic radii of solutes ranging from 0.1 to 300 nm [22,23,24]. TDA offers several advantages, including low sample consumption (requiring only a few nL per injection), no need for sample filtration (insensitive to dust, no chromatographic column to protect from clogging), being an absolute method (no size calibration required), rapid analysis, and automation. The interactions between substrate and ligand at equilibrium can also be studied using TDA [25,26,27,28]. In the case of fast kinetic interactions, where entities exchange rapidly during the analysis, the resulting Taylorgram shows a simple Gaussian peak [28], with peak broadening (variance) that increases with increasing fraction of substrate-ligand. It is thus possible to determine the binding constant by observing the changes in diffusivity or size with varying ligand/substrate ratios [25]. In the case of slow kinetic interactions, the presence of two distinguishable Gaussian peaks in the Taylorgram, one for each population (free ligand and complex), indicates that the dissociation kinetics of the substrate-ligand are slow relative to the timescale of the analysis [26,27]. In that situation, the deconvolution of the Taylorgram provides information on both the size of the complex and the relative proportion of free ligand, allowing to plot the isotherm of adsorption of the ligand onto the substrate [26].

This study investigates the interaction of neutral and negatively charged GBCAs with collagen at physiological pH using TDA. Frontal analysis continuous capillary electrophoresis (FACCE), a separation method used to study substrate/ligand interactions based on differences in electrophoretic mobility between interacting partners [29,30,31,32,33], was also used to confirm the results obtained by TDA.

## 2. Results and Discussion

### 2.1. Workflow Analysis

**Figure 1:**
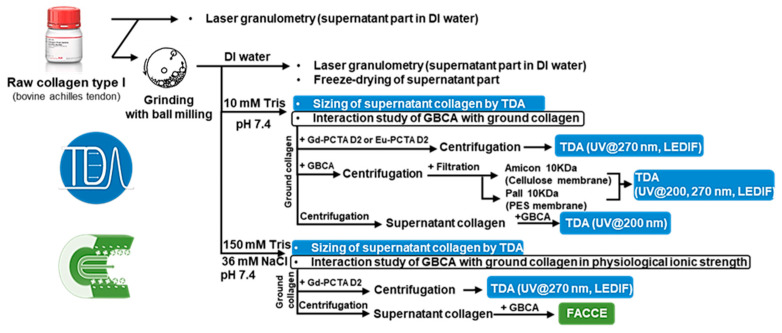
Presentation of the workflow analysis for the study of GBCA/collagen interactions.

The workflow analysis of this work is presented in Figure 1 to clearly identify the key steps involved in this study, including grinding, buffering, centrifugation, with or without filtration and analysis by TDA or FACCE. Starting with raw type I collagen, the size distributions of collagen suspensions in water were measured using laser granulometry for both raw and ground collagen samples. TDA provided additional characterization of the soluble part of collagen in the supernatant, determining the average hydrodynamic radius (*R_h_*) in both 10 mM tris and 150 mM tris buffer pH 7.4. The interactions of GBCA with ground collagen, after centrifugation alone or centrifugation and ultra-filtration, or with the soluble supernatant collagen were studied by TDA in tris 10 mM pH 7.4. Since TDA can size the soluble nano-objects that are in the range of 0.1 to 300 nm. If GBCA interacts with collagen, its apparent size would increase. Two cases can occur: in the case of fast kinetics of interaction, the apparent sizes of GBCA should increase and correlate with the bounded fraction of GBCA in the mixture [25]. In the case of interactions with slow kinetics, both free and bound GBCA fractions can be quantified and sized by deconvolution of the Taylorgram using two Gaussian fittings (see, e.g., [26]). 

In our first attempt to study the interaction between GBCA and ground collagen, the remaining soluble part of collagen contained in the supernatant after centrifugation of the equilibrated mixture masked the GBCA response using UV absorbance at 200 nm (or fluorescent detection). Specific detection of GBCA was needed, but only some of them responded at 270 nm (where the response of collagen was negligible), namely Gd-PCTA D2 and Eu-PCTA D2. The use of centrifugal ultra-filtration of the mixture was also investigated to remove most of the soluble collagen part from the mixture to improve the quantification of free GBCA. Alternatively, we examined GBCA interactions with the soluble part of ground collagen (supernatant collagen) by realizing collagen centrifugation before adding the GBCA. This latter method is advantageous as the collagen sample is more homogeneous in the solution and does not require filtration for TDA or FACCE analysis (no risk of capillary clogging). Finally, the interaction of GBCA with collagen in 150 mM tris with 36 mM NaCl (165 mM ionic strength, pH 7.4) was investigated using FACCE and TDA.

### 2.2. Presentation of Collagen and GBCA Sample

#### 2.2.1. Preparation and Characterization of Collagen

Raw type 1 collagen from bovine achilles tendon is mainly non-soluble in water or in buffer solution at physiological pH. The raw collagen is non-homogenous in size, even at a macroscopic scale, as evidenced by the presence of large particles highlighted in the red circle in Figure S1A. This creates a significant challenge: as the interactions with GBCA would occur mainly at the surface of the collagen particles, it raises concerns about the reproducibility of specific surface areas between samples. To address this issue, the raw collagen was ground to achieve a more homogeneous starting material (see Section 2.1). Moreover, after mixing the ground collagen with buffer (see Section 2.3), supernatant collagen was prepared by centrifugation and used as a starting material to study the interactions with GBCA. Freeze-drying the supernatant in deionized water (Figure S1D) allowed the quantification of collagen concentration in the supernatant solution, determined to be approximately 3.65 ± 0.53 g/L (4.87% of the initial concentration of raw collagen based on three replicates). Despite the inherent solubility limitations of collagen and the associated material loss, the reasonable cost of raw bovine achilles tendon collagen and the small experimental volumes used made this approach feasible for interaction studies.

##### Laser Diffraction Granulometry

**Figure 2:**
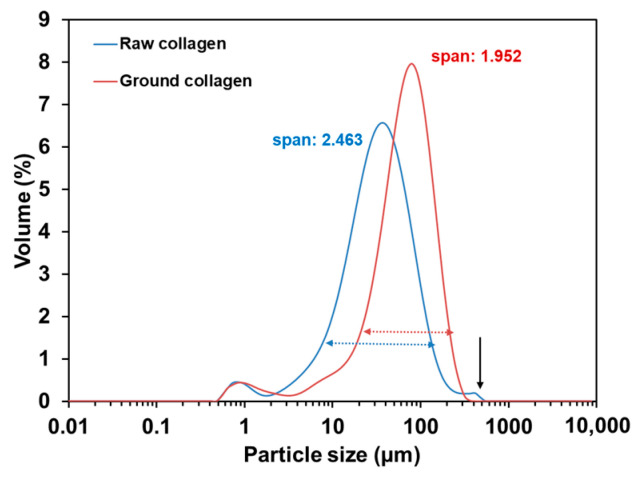
Laser diffraction granulometry of raw collagen and ground collagen 5 g/L suspended in water. The span number is a parameter that indicates the width of particle size distribution. For raw collagen, a significant fraction of the material flocculated before the laser analysis.

Figure 2 shows the particle size distributions of the original collagen supernatant (blue) compared with the ground collagen supernatant (red). The results indicate that the large particles, approximately 300–600 µm (indicated by the black arrow), were effectively eliminated, while the smaller particles remained unchanged. Also, the fraction of macroscopic collagen (~mm and sub-mm range) which rapidly sedimented before laser diffraction analysis, was drastically decreased by the grinding (see also Figure S1). The span value in the graph, which describes the width of the particle size distribution, decreased, indicating a more homogeneous size distribution. Therefore, grinding using a vibratory ball mill effectively reduced macroscopic particles, yielding a more uniform sample (lower span value, lower flocculation, and lower macroscopic fraction).

##### Size of Collagen by Taylor Dispersion Analysis

TDA is a sizing technique based on the analysis of the dispersion of a sample band (or sample front in the frontal mode) under a laminar flow in a narrow bore capillary [24]. The dispersion of the sample band (or front) directly depends on the diffusion coefficients of the analytes that are present in the sample. This method can be used to size nano-objects in the 0.1–300 nm range [22] or to study interactions [25,26]. The equations used to determine the diffusion coefficient and the hydrodynamic radius are presented in Section 1 of SI.

**Figure 3:**
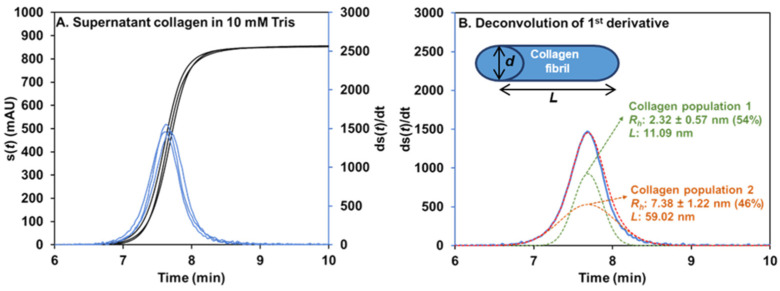
Frontal Taylorgrams (black) and its 1st derivative (blue) of 3.65 g/L supernatant collagen in 10 mM tris pH 7.4 with UV detection at 200 nm (**A**). Deconvolution of 1st derivative Taylorgram by Gaussian fitting with two Gaussian curves (**B**). Fit is plotted as a red dotted line. Experimental conditions: fused silica capillary of 65 cm total length (56.5 cm to UV detector) × 50 µm i.d. eluent: 10 mM tris buffer (pH 7.4). Mobilization pressure: 100 mbar. Experiments were performed at 37 °C. Supernatant collagen was prepared following the procedure described in Section 2.3.

Frontal Taylorgrams (three repetitions) of the supernatant collagen in 10 mM tris buffer pH 7.4 are displayed in Figure 3A. Collagen exhibits a very strong response in UV at 200 nm (~ 850 mAU for a 3.65 g/L solution). The first derivatives of the elution profiles are also presented (blue line in Figure 3A), as they provide a clearer visualization of the signal dispersion and symmetry. Taylorgrams of the supernatant collagen in 150 mM tris buffer pH 7.4 are shown in Figure S2, and displayed some spikes on the elution profile. This may result from the increased aggregation of collagen at higher ionic strength. *R_h_* of collagen was determined using Equations (S2) and (S3) given in Section S1 of the Supporting Information. Signal deconvolution (Figure 3B) revealed two populations of different sizes in solution, which are approximately equally represented in mass. In 10 mM tris buffer, *R_h_* of collagen is 2.32 ± 0.57 nm (54%) and 7.38 ± 1.22 nm (46%). Larger sizes of supernatant collagen were found in 150 mM tris with *R_h_* = 3.37 ± 0.17 (48%) and 18.06 ± 1.99 (52%). The supernatant solution in 150 mM tris is more turbid compared to that in water (see Figure S1C). This turbidity is visible to the naked eye but difficult to capture in a photo. The proportions between the two populations obtained from the peak area correspond to mass fraction since the UV detector is sensitive to the mass concentration [34]. Similarly, it was previously demonstrated that the *R_h_* obtained by TDA was a weight-average value within a given population, which may necessarily be more or less polydisperse [34].

According to the literature, a single collagen fibril has a diameter (*d*) of approximately 1.5 nm and a length (*L*) of about 300 nm [35]. Assuming that the soluble collagen populations have the same diameter (1.5 nm), we can estimate the length of supernatant collagen fibril in each population from the hydrodynamic radius (*R_h_*) using the equation described by Tirado et al. [36,37]. This *R_h_* estimation considers that the experimental diffusion coefficient determined by TDA corresponds to an average over all the orientations of the molecule relative to the flow direction. In 10 mM tris buffer, the calculated lengths of collagen fibrils are 11 and 59 nm for populations 1 and 2, respectively. The length of 150 mM tris buffer is 19.8 and 185 nm for populations 1 and 2, respectively. Of course, collagen fibrils may also partially aggregate into thicker structures and, therefore, other *d*, *L* combinations are possible, leading to similar *R_h_* values. Still, TDA is a remarkable technique to characterize the size distribution of soluble fractions of collagen in a given buffer.

#### 2.2.2. Characteristics of GBCA and Collagen Detections in UV and Fluorescence

The UV and fluorescence spectra of the GBCA were recorded at the same concentration of 5.0 mM, and the spectrum of supernatant collagen at 3.65 g/L was overlaid for comparison (see Figure S3). From these spectra, a comparison of the UV and fluorescence detection characteristics is provided in Table 1. It can be seen that Gd-DTPA-BMA, Gd-BOPTA, Gd-HP-DO3A, Gd-BT-DO3A, and Gd-DOTA exhibited UV responses in the same region and with lower absorbance than that of the supernatant collagen at the experimented concentrations. However, Gd-PCTA D2 and Eu-PCTA D2 presented a maximum absorption at 270 nm, characteristic of the pyridyl chromophore [38], and at which the supernatant collagen was transparent. In Figure S3B, the emission spectra of GBCA are presented at an excitation wavelength of 275 nm. The results indicate that all GBCA exhibited an emission peak in the same region as collagen, except for Eu-PCTA D2, which showed a strong double peak attributed to the Eu^3+^ complex at 595–620 nm ([39]).

### 2.3. Interaction Between Collagen and GBCA in 10 mM Tris pH 7.4

#### 2.3.1. GBCA Interaction with Ground Collagen by TDA After Centrifugation

**Figure 4:**
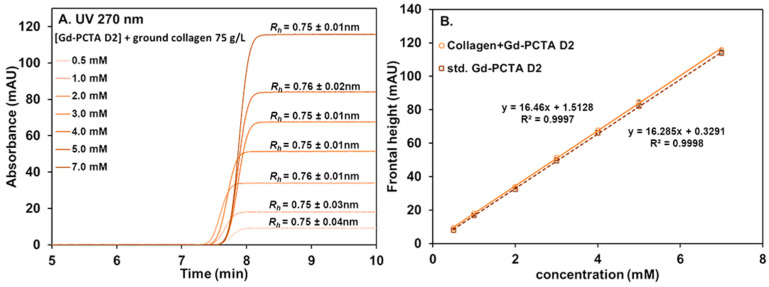
Frontal Taylorgrams of Gd-PCTA D2 (0.5–7.0 mM) in the presence of ground collagen (75 g/L) and after centrifugation (**A**). Linear calibration curves of Gd-PCTA D2 obtained by frontal TDA in the absence (dotted line) and in the presence of ground collagen after centrifugation (solid line) at 270 nm (**B**). Experimental conditions: fused silica capillary of 65 cm total length (56.5 cm to UV detector) × 50 µm i.d. eluent: 10 mM tris buffer (pH 7.4). Mobilization pressure: 100 mbar. UV detection at 270 nm. Incubation of mixture: 37 °C 1000 rpm for 4 h. TDA experiments were performed at 37 °C.

To study the interaction between GBCA and ground collagen, 37.5 mg of ground collagen was added to 500 µL of GBCA at varying concentrations. This mixture cannot be directly injected for TDA analysis because collagen can clog the capillary. Therefore, centrifugation (at 12,000 rpm for 3 min) was required to remove the insoluble collagen part. As discussed in the detection of GBCA section, only Gd-PCTA D2 and Eu-PCTA D2 can be studied using selective detection UV at 270 nm. Additionally, LEDIF (λ_ex_ 275 nm, λ_collection_ higher than 480 nm) was used for Eu-PCTA D2. Figure 4 displays the frontal Taylorgrams obtained for Gd-PCTA D2 after the interaction with ground collagen at UV 270 nm and the corresponding calibration plots (plain line, Figure 4B) in comparison with the GBCA standards without collagen (dotted line, Figure 4B). It clearly shows that all initially introduced Gd-PCTA D2 recovered, even after centrifugation, since no decrease in frontal height was observed after the interaction. Therefore, we can conclude that the GBCA was not trapped by the collagen eliminated during the centrifugation. The size of Gd-PCTA D2 reported in the previous study is *R_h_* = 0.70 nm [40]. A slight increase in Gd-PCTA D2 size (0.75 nm) was observed, but it could be attributed to a slight viscosity increase (soluble collagen, 9.73 × 10^−4^ Pa s vs. 8.9 × 10^−4^ Pa s for 10 mM tris buffer). This effect could not be attributed to interaction with collagen since the apparent size of the GBCA did not change with the increasing concentration of Gd-PCTA D2. Therefore, we can conclude that no interaction could be observed under these conditions. The same result was obtained with Eu-PCTA D2, as shown in Figure S4 (UV 270 nm and LEDIF detections with λ_collection_ higher than 480 nm). There was no significant decrease in Eu-PCTA D2 concentration for both detection modes in the presence of collagen.

Similar results were obtained in (150 mM tris buffer with 36 mM NaCl pH 7.4) as demonstrated by the frontal Taylorgrams of Gd-PCTA D2 in the absence and in the presence of collagen presented in Figure S5.

#### 2.3.2. On the Use of Centrifugal Filter for the Study of GBCA/Collagen Interactions

**Figure 5:**
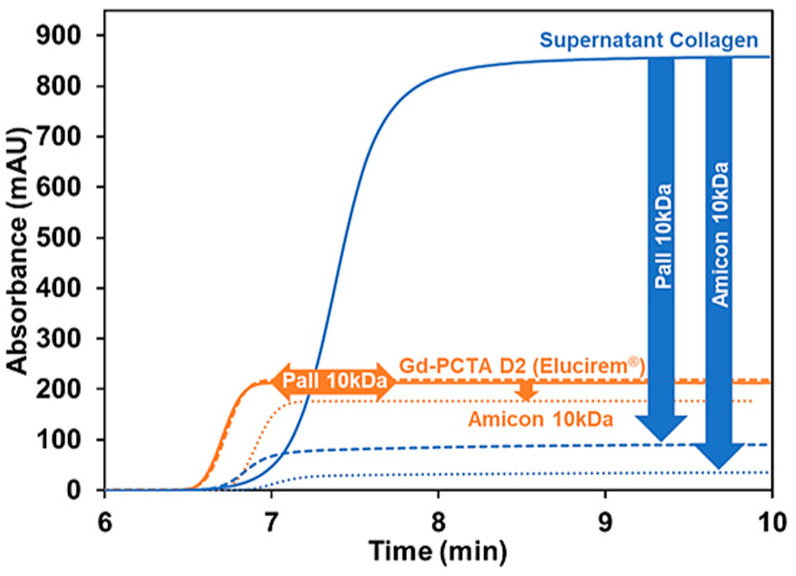
Frontal Taylorgrams showing the potential retention of supernatant collagen (3.65 g/L, in blue) and Gd-PCTA D2 (2.5 mM, in orange) before and after filtration using Amicon^®^ and Pall^®^ devices with MWCO of 10 kDa. Experimental conditions: fused silica capillary of 65 cm total length (56.5 cm to UV detector) × 50 µm i.d. eluent: 10 mM tris buffer (pH 7.4). Mobilization pressure: 100 mbar. UV detection at 200 nm. Incubation of mixture: 37 °C 1000 rpm for 4 h. Sample volume: 60 µL. TDA experiments were performed at 37 °C.

Collagen has a very high response in UV and LEDIF, making it impossible to detect GBCA in the mixtures if GBCA responds only in UV at 200 nm and by fluorescence with λ_em_ 300–400 nm, as the collagen masks all other responses. To better remove collagen from the mixture and prevent capillary clogging, filtration using centrifugal filters was investigated, in agreement with previous work that reported the use of Amicon^®^ filtration [18]. Two types of filters with a molecular weight cut-off (MWCO) of 10 kDa: Amicon^®^ (cellulose membrane) and Pall^®^ (Polyethersulfone membrane), were investigated. Before injecting the equilibrated mixture in the filtration device, the supernatant collagen at 3.65 g/L in tris 10 mM was analyzed by TDA before and after filtration using Amicon^®^ or Pall^®^ filtration setups in 10 mM tris solution. As displayed in blue in Figure 5, a significant fraction of collagen can still pass through both filters. The hydrodynamic radius of the soluble filtrated collagen is about 1.5–2 nm for both filters (i.e., lower than the supernatant collagen in the same medium, 2.3 nm (54%) and 7.4 nm (46%)). The filtrated collagen solutions using Amicon^®^ and Pall^®^ showed UV responses at 200 nm of 35 mAU and 80 mAU, respectively, which still represent a significant contribution compared to the absorbance of 2.5 mM Gd-PCTA D2 at 215 mAU (see Figure 5).

Surprisingly, the Amicon^®^ filter retained a significant fraction of GBCA, as demonstrated in Figure 5 for Gd-PCTA D2, since the filtrated solution showed a lower response than the initial concentration. No measurable retention could be observed on the Pall^®^ setup. Table S1 shows similar observations for the other GBCA tested. The signal loss ranges from 8.8% to 50% for the Amicon^®^ filter at 2.5 mM GBCA concentration in 10 mM tris buffer, depending on the GBCA. Thus, the cellulose-type membrane of the Amicon^®^ device is not appropriate for studying the interaction of GBCA.

The retention of GBCA on the Pall^®^ filter was also tested (see Table S1). There was no decrease in the frontal signal after filtration of the GBCA solution, but the Pall^®^ filter also failed to retain all the soluble fractions of collagen. Therefore, centrifugal filtration was no longer considered for the study of GBCA interaction with collagen.

#### 2.3.3. Study of Interaction of GBCA and Supernatant Collagen Having Similar UV Responses by TDA

**Figure 6:**
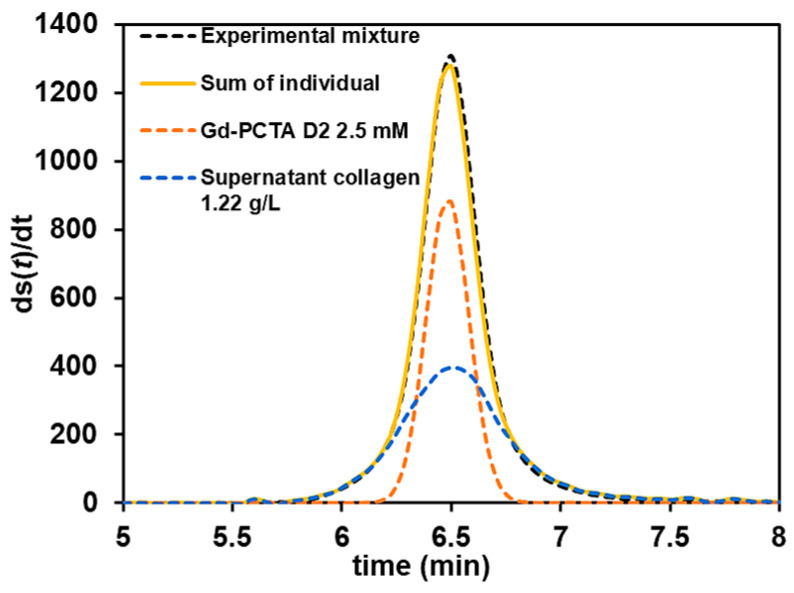
The first derivative of the experimental mixture (black trace) and the sum of the individual (yellow) of 1.22 g/L supernatant collagen (blue) and 2.5 mM Gd-PCTA D2 (orange). Experimental conditions: fused silica capillary of 65 cm total length (56.5 cm to UV detector) × 50 µm i.d. eluent: 10 mM tris buffer, pH 7.4. Mobilization pressure: 100 mbar. UV detection at 200 nm. Incubation of mixture: 37 °C 1000 rpm for 4 h. Sample volume: 60 µL. TDA experiments were performed at 37 °C.

In this section, another method to study the GBCA/collagen interaction by mixing GBCA and supernatant collagen at concentrations that provide equivalent frontal heights at UV 200 nm is proposed. The concentrations of GBCA were set at 2.5 mM for Gd-PCTA D2, Gd-DTPA-BMA, and Gd-BOPTA, and at 4.0 mM for the low-responding UV, Gd-DOTA, Gd-BT-DO3A, and Gd-HP-DO3A. The concentration of supernatant collagen providing a similar UV response is reported in Table S2. We compared the first derivative signal between the sum of individual signals and the experimental mixture at the same final concentrations. Figure 6 illustrates the results of 2.5 mM Gd-PCTA D2 with 1.22 g/L supernatant collagen. It is obvious that the size and response of the mixture Gd-PCTA D2 and supernatant collagen after the interaction (yellow line) did not change from the sum of the individual introduced solutions (black dotted line). This result clearly demonstrates that there was no interaction between Gd-PCTA D2 and collagen. If interaction had occurred, either a part of the GBCA population would have decreased to the benefits of collagen or only one Gaussian peak would have been observed in the case of fast kinetics interactions. None of these cases was observed; therefore, we confirm the absence of interaction, at least in the investigated conditions. Similar results were obtained for the other GBCA, as presented in Table S2.

### 2.4. Interaction Between Collagen and GBCA in 150 mM Tris pH 7.4

#### Study of GBCA/Collagen Interactions Using Frontal Analysis Continuous Capillary Electrophoresis (FACCE)

**Figure 7:**
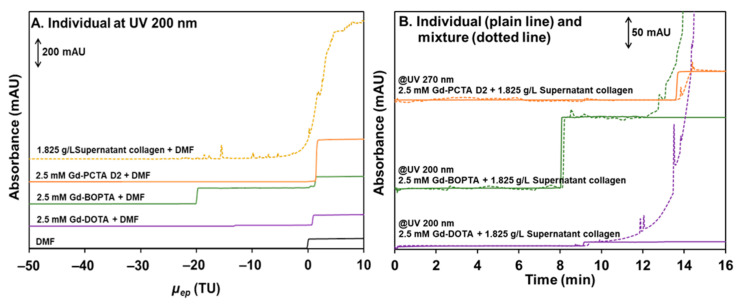
Frontal electropherograms showing the electrophoretic mobility of individual Gd-PCTA D2, Gd-BOPTA, Gd-DOTA, and supernatant collagen in the effective mobility scale (**A**). Timescale frontal electropherograms of 2.5 mM Gd-PCTA D2, Gd-BOPTA, Gd-DOTA standards (plain lines) and their mixtures in the presence of 1.825 g/L supernatant collagen (dotted lines) (**B**). Experimental conditions: PDADMAC coated capillary of 65 cm total length (56.5 cm to UV detector) × 50 µm i.d. eluent: 150 mM tris with 36 mM NaCl buffer (pH 7.4). UV detection at 200 and 270 nm. Incubation of mixture: 37 °C 1000 rpm for 4 h. Applied voltage: −15 kV (from inlet) for standard GBCA and applied co-pressure +50 mbar (from inlet) for supernatant collagen and the mixture. Sample volume: 120 µL. FACCE experiments were performed at 37 °C.

Finally, the FACCE method was used to study the interaction. In FACCE, the idea is to separate the free GBCA from the GBCA/collagen-associated entity by continuous electrophoresis. GBCA and collagen (or the associated entity) are thus separated according to their charge-to-size ratio. FACCE is a frontal mode; therefore, the separated solutes are detected as successive fronts according to their respective electrophoretic mobilities. As confirmed by the electrophoretic mobility shown in Figure 7A, only Gd-BOPTA and Gd-DOTA are negatively charged and can be effectively separated from the almost neutrally charged supernatant collagen. Therefore, free Gd-BOPTA and Gd-DOTA can be quantified in their equilibrated mixtures with supernatant collagen by UV detection at 200 nm and by LEDIF for Gd-DOTA. The other GBCA is neutral and cannot be separated from supernatant collagen. However, Gd-PCTA D2 was selectively detected at UV 270 nm since collagen does not respond at this wavelength. The interaction between 2.5 mM GBCA and 1.825 g/L supernatant collagen was compared with the response of the standard GBCA solution at the same concentration. The frontal heights of Gd-BOPTA and Gd-DOTA after interaction were not different from the standard GBCA solutions at the same concentrations, either in UV detection at 200 nm (Figure 7B) or LEDIF (Figure S6). Additionally, the response of Gd-PCTA D2 at UV 270 nm (Figure 7B) remained unchanged except for a shift in time due to the viscosity effect. Therefore, with supernatant collagen, FACCE also confirmed the absence of measurable interactions between Gd-BOPTA, Gd-DOTA, and Gd-PCTA D2.

## 3. Materials and Methods

### 3.1. Materials

The structures of the GBCAs used in this study are listed in Table 2. These include Gd-DTPA-BMA (Gadodiamide, Omniscan^®^, GE-Healthcare, Chicago, IL, USA), Gd-BOPTA (Gadobenate dimeglumine, MultiHance^®^, Bracco, Milan, Italy), Gd-HP-DO3A (Gadoteridol, ProHance^®^, Bracco), Gd-BT-DO3A (Gadobutrol, Gadovist^®^, Bayer Healthcare, Berlin, Germany), Gd-DOTA (Gadoterate meglumine, Dotarem^®^, Guerbet, Villepinte, France), Gd-PCTA D2 (Gadopiclenol, Elucirem^®^, Guerbet), and Eu-PCTA D2. The europium analogue complex of Gadopiclenol (Eu-PCTA D2) was prepared by replacing gadolinium with europium chloride during the complexation step. All GBCA solutions were diluted to the desired concentration either in 10 mM tris buffer pH 7.4 or in 150 mM tris buffer with 36 mM NaCl pH 7.4. Tris(hydroxymethyl)aminomethane (tris) and Poly(diallyldimethyl ammonium chloride) (PDADMAC), Mw 400–500 kDa, were purchased from Sigma Aldrich, Saint-Quentin-Fallavier, France.

Type I Collagen from bovine achilles tendon (Sigma Aldrich, Saint-Quentin-Fallavier, France), pI 7.0–8.0, Mw about 250–300 kDa, was ground using a vibratory ball mill machine MM200 (Retsch, Eragny sur Oise, France, Figure S7) for a better homogeneity in size and surface area. A 5 g sample of raw collagen was introduced in a stainless-steel jar with one stainless-steel ball (1 cm in diameter) and subsequently shaken at 25 Hz for 4 min.

### 3.2. Laser Diffraction Granulometry

Laser diffraction granulometry was performed to measure the particle size distribution of raw and ground collagen in water using Master Sizer 2000 (Malvern Instruments, Palaiseau, France). Both raw and ground collagen (5 g/L) were suspended in water using a vortex mixer (Phoenix Instrument, Garbsen, Germany). The larger collagen particles were allowed to settle down for approximately 30 s and then only the supernatant part was carefully loaded to measure the size distribution of the suspended collagen.

### 3.3. Preparation of Supernatant Collagen

A 75 mg sample of ground collagen was suspended in 1 mL buffer solution and incubated using a thermal shaker at 37 °C 1000 rpm for 4 h (VMR^®^, Rosny-sous-Bois, France). After that, the suspension was centrifuged at 12,000 rpm for 3 min. The supernatant solution was used to study interaction with GBCA further. Freeze-drying the precipitated collagen enables the estimation of the collagen concentration in the supernatant.

### 3.4. Interaction Study of GBCA with Collagen

The interaction was studied by mixing 37.5 mg of ground collagen with 500 µL of GBCA solutions at different concentrations (0.5–7.0 mM) in 10 mM tris or 150 mM tris buffer in a 500 µL Eppendorf. The mixture was incubated on a thermal shaker at 37 °C, 1000 rpm, for 4 h. This experimental condition was adapted from Guidolin et al. [18]. Subsequently, a centrifugation step at 12,000 rpm for 3 min (with/without an ultra-filtration on 10 kDa) was used to remove collagen from the mixture before quantifying GBCA by Taylor dispersion analysis (TDA) or frontal analysis continuous capillary electrophoresis (FACCE). Similarly, ~0.08–1.22 g/L supernatant collagen in the buffer was mixed with GBCA at a final concentration of 2.5–4.0 mM (1:1 *v*/*v*) in the buffer.

### 3.5. Taylor Dispersion Analysis (TDA)

TDA experiments were conducted on an Agilent 7100 CE system (Waldbronn, Germany) equipped with a diode array UV detector and a Zetalif LED-induced fluorescence detector (LEDIF) from Adelis (Toulouse, France). The experimental condition was adapted from the previous TDA study on GBCA [40]. Analyses were carried out in a bare fused-silica capillary (Polymicro technologies, Phoenix, AZ, USA) with a 50 µm i.d. × 65 cm total length. The LEDIF and UV detection windows were positioned at 44 cm and 56.5 cm from the inlet, respectively.

Capillaries were conditioned before analysis by flushing (at 1 bar) with water for 10 min followed by the background electrolyte (BGE) for 10 min. BGE was either 10 mM tris or 150 mM tris with 36 mM NaCl buffer at pH 7.4. A 60 µL sample was placed into a 250 µL polypropylene vial (Agilent, Waldbronn, Germany). Short electrodes were used to prevent contact with the sample solution. Samples were continuously introduced at 100 mbar from the inlet side of the capillary. Between runs, the capillary was rinsed by flushing with the BGE for 5 min. UV detection was carried out at 200 and 270 nm with a bandwidth of 4 nm. The fluorescent signals were measured at an excitation wavelength (λ_ex_) of 275 nm, and the signals were collected (λ_collection_) from 300 to 450 nm or above 480 nm, depending on the filter used. All experiments were performed at 37 °C. The temperature of the carousel holding BGE and sample vials was controlled using a thermostatic bath set at 37 °C (Instrumat, Moirans, France). Signal acquisition was carried out using Chemstation software (B.04.02 SP1), and then exported to Microsoft Excel (version 2307) for subsequent data treatment as described in SI.

### 3.6. Frontal Analysis Continuous Capillary Electrophoresis (FACCE)

A 50 µm i.d. × 65 cm total length bare fused silica capillary was coated with 0.2% *w*/*w* PDADMAC in 2 M NaCl using the protocol described by Lounis et al. [41]. For FACCE experiments, BGE consisted of 150 mM tris with 36 mM NaCl at pH 7.4 (165 mM ionic strength). DMF was used as a neutral marker to evaluate the mobility and charge of collagen and GBCAs. A volume of 120 µL of sample was placed into a 250 µL polypropylene vial at the capillary inlet and was introduced continuously and electrokinetically in the capillary. Standard electrodes were used for FACCE analysis to ensure electrical contact with the sample. A continuous negative polarity voltage of –15 kV was applied for standard GBCA solutions and –15 kV with an additional co-pressure +50 mbar for supernatant collagen and the GBCA/collagen mixtures. The outlet vial contained 450 µL of BGE in a 1 mL polypropylene vial. The solution levels in both the inlet and outlet vials were maintained at the same height. The co-pressure allowed the entrance and the quantification of the free GBCA in the mixture, compensating for the higher viscosity of the supernatant collagen solution compared to the buffer.

## 4. Conclusions

In this work, we investigated the use of TDA and FACCE to study the possible interactions between GBCA and collagen. Due to the non-solubility of collagen in physiological pH conditions, this study is challenging due to high heterogeneity of the collagen sample in such conditions. To that respect, grinding the collagen sample could help to homogenize the sample by reducing the fraction of large macroscopic collagen (sub-millimeter range). For better reproducibility in interaction studies, we also proposed investigating the interactions with collagen’s soluble (or suspended) supernatant fraction. TDA characterized This homogeneous fraction in size and contained two populations in the nm range (*R_h_* = 2.3 and 7.4 nm), with larger sizes when the ionic strength increases (3.4 and 18.1 nm). The use of filtration to remove the collagen should be considered with great care since we demonstrated that the GBCA can be retained by the filter (especially with the cellulose filters Amicon^®^ MWCO 10 kDa). This filter retention can introduce artefacts in the interaction studies. Moreover, some of the soluble collagen of low size (~1.5–2 nm) is not retained by the filter. The interaction study performed by TDA between GBCA and ground collagen (or supernatant collagen) showed no significant interactions between the GBCA (Gd-PCTA D2, Gd-DTPA-BMA, Gd-BOPTA, Gd-DOTA, Gd-BT-DO3A, and Gd-HP-DO3A) and collagen. Similar conclusions were obtained by frontal analysis continuous capillary electrophoresis (FACCE) either for negatively charged GBCA (Gd-BOPTA and Gd-DOTA) or neutral GBCA (Gd-PCTA D2) with no changes in free GBCA concentration in the presence of supernatant collagen. Overall, our study underlines that, under the tested conditions, GBCA does not interact significantly with collagen, whether ground or in supernatant forms.

## Figures and Tables

**Table 1:** Comparison of characteristic UV absorbance and fluorescence emission (λ_ex_ 275 nm) between GBCA and collagen.

Sample	Charge at pH 7.4	UV (nm)		LEDIF Emission (nm) λ_ex_ 275 nm
Gd-DTPA-BMA (Omniscan^®^)	neutral	190–218	–	307–320
Gd-BOPTA (Multihance^®^)	negative	190–220	–	–
Gd-HP-DO3A (Prohance^®^)	neutral	190–208	–	307–320
Gd-BT-DO3A (Gadovist^®^)	neutral	190–208	–	307–320
Gd-DOTA (Dotarem^®^)	negative	190–208	–	307–320
Gd-PCTA D2 (Elucirem^®^)	neutral	190–225	250–280	300–400
Eu-PCTA D2	neutral	190–230	250–280	595–620
Supernatant collagen	neutral	190–220	–	300–400

**Table 2:** Structure of complexes.

Neutral Complexes (at pH 7.4)	Negatively Charged Complexes (at pH 7.4)
Gd-DTPA-BMA (Gadodiamide) 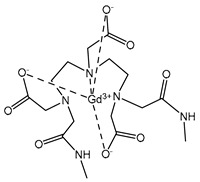	Gd-BOPTA (Gadobenate) 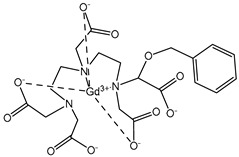
Gd-HP-DO3A (Gadoteridol) 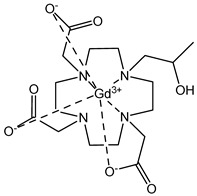	Gd-DOTA (Gadoterate) 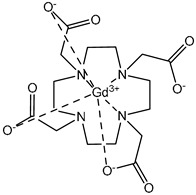
Gd-BT-DO3A (Gadobutrol) 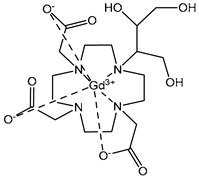	
Gd-PCTA D2 (Gadopiclenol) 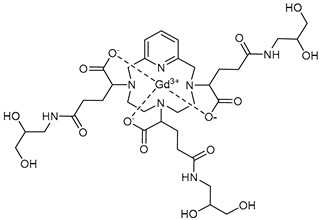	
Eu-PCTA D2 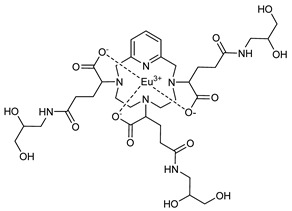	

## Data Availability

Data is contained within the article or Supplementary Material.

## Supplementary Materials

The following supporting information can be downloaded at: https://www.mdpi.com/article/10.3390/ph17121633/s1, Section S1: TDA Data treatment; Section S2: Tables and figures; Figure S1: Photos of raw collagen 5 g/L in water (A), ground collagen 75 g/L in water (B) and ground collagen 75 g/L after centrifugation in 150 mM tris-HCl buffer (C, left) and in ultrapure water (C, right); Freeze dried supernatant collagen in 150 mM tris-HCl buffer (D, left) and in water (D, right); Figure S2: Frontal Taylorgrams (black) and 1st derivative (blue) of supernatant collagen in 150 mM tris with 36 mM NaCl pH 7.4 UV detection at 200 nm (A). Deconvolution of 1st derivative Taylorgram with 2 gaussian curves (B); Figure S3: UV-Visible spectra (A) and emission spectra (B, λ_ex_ 275 nm) of 5 mM GBCA and supernatant collagen; Figure S4: Frontal Taylorgrams of Eu-PCTA D2 (0.5–5.0 mM) in the presence of ground collagen (75 g/L) and after centrifugation in UV mode at 270 nm (A) and using LEDIF detection (C). Linear calibrations of Eu-PCTA D2 obtained by TDA in frontal mode in the absence (dotted line) and in the presence (solid line) of ground collagen at UV 270 nm (B) and LEDIF detection (D); Figure S5: Frontal Taylorgrams of Gd-PCTA D2 (1.25 and 2.5 mM) after interaction with ground collagen (75 g/L) and centrifugation (dotted lines). Standard of Gd-PCTA D2 (solid line); Table S1: Frontal Taylorgrams of GBCA before (plain line) and after filtration (dotted line) with UV detection at 200 nm testing the retention of GBCA on the centrifugal filter (Amicon and Pall); Table S2: Frontal Taylorgrams and its corresponding first-derivative obtained for GBCA alone (first column), supernatant collagen (second column), experimental mixture between GBCA and supernatant collagen at the same final concentration as individual (third column), and overlay of all the Taylorgrams showing the absence of significant interactions; Figure S6: Time-scale frontal electropherograms obtained by LEDIF detection of 2.5 mM Gd-DOTA standard (plain lines) and their mixtures in the presence with 1.825 g/L supernatant collagen (dotted lines); Figure S7: Vibratory ball mill machine MM200;.
