# Supplementary material for: Study of Interactions Between Gadolinium-Based Contrast Agents and Collagen by Taylor Dispersion Analysis and Frontal Analysis Continuous Capillary Electrophoresis
Source: Pharmaceuticals (Basel). 2024 Dec 5;17(12):1633. doi: 10.3390/ph17121633 (PMC11728588; doi:10.3390/ph17121633)
Supplement: Supplementary file 1 [file pharmaceuticals-17-01633-s001.zip › pharmaceuticals-3304125-supplementary.pdf]

Supporting Information

Study of Interactions Between Gadolinium-Based Contrast Agents and Collagen by Taylor Dispersion Analysis and Frontal Analysis Continuous Capillary Electrophoresis#

Chutintorn Somnin [1], Joseph Chamieh [1,*], Laurent Leclercq [1], Christelle Medina [2], Olivier Rousseaux [2] and Hervé Cottet [1,*]

[1] IBMM, University of Montpellier, CNRS, ENSCM, 34095 Montpellier, France; chutintorn.somnin@etu.umontpellier.fr (C.S.); laurent.leclercq@umontpellier.fr (L.L.)
[2] GUERBET, Research and Innovation, 16 rue Jean Chaptal, 93600 Aulnay Sous Bois, France; christelle.medina@guerbet.com (C.M.); olivier.rousseaux@guerbet.com (O.R.)
* Correspondence: joseph.chamieh@umontpellier.fr (J.C.); herve.cottet@umontpellier.fr (H.C.); Tel.: +33-44-879-2179 (J.C.); +33-44-879-2196 (H.C.)

TABLE OF CONTENTS

Section 1: TDA Data treatment 3

Section 2: Tables and figures...4

Figure S1. Photos of raw collagen 5 g/L in water (A), ground collagen 75 g/L in water (B) and ground collagen 75 g/L after centrifugation in 150 mM Tris-HCl buffer (C, left) and in ultrapure water (C, right); Freeze dried supernatant collagen in 150 mM Tris-HCl buffer (D, left) and in water (D, right). 4

Figure S2. Frontal taylorgrams (black) and 1st derivative (blue) of supernatant collagen in 150 mM Tris with 36 mM NaCl pH 7.4 UV detection at 200 nm (A). Deconvolution of 1st derivative taylorgram with 2 gaussian curves (B). 5

Figure S3. UV-Visible spectra (A) and emission spectra (B, λ_{ex} 275 nm) of 5 mM GBCA and supernatant collagen 5

Figure S4. Frontal taylorgrams of Eu-PCTA D2 (0.5 – 5.0 mM) in the presence of ground collagen (75 g/L) and after centrifugation in UV mode at 270 nm (A) and using LEDIF detection (C). Linear calibrations of Eu-PCTA D2 obtained by TDA in frontal mode in the absence (dotted line) and in the presence (solid line) of ground collagen at UV 270 nm (B) and LEDIF detection (D). 6

Figure S5. Frontal taylorgrams of Gd-PCTA D2 (1.25 and 2.5 mM) after interaction with ground collagen (75 g/L) and centrifugation (dotted lines). Standard of Gd-PCTA D2 (solid line). 7

Table S1. Frontal taylorgrams of GBCA before (plain line) and after filtration (dotted line) with UV detection at 200 nm testing the retention of GBCA on the centrifugal filter (Amicon and Pall) 8

Table S2. Frontal taylorgrams and its corresponding first-derivative obtained for GBCA alone (first column), supernatant collagen (second column), experimental mixture between GBCA and supernatant collagen at the same final concentration as individual (third column), and overlay of all the taylorgrams showing the absence of significant interactions. 9

Figure S6. Time-scale frontal electropherograms obtained by LEDIF detection of 2.5 mM Gd-DOTA standard (plain lines) and their mixtures in the presence with 1.825 g/L supernatant collagen (dotted lines). 10

Figure S7. Vibratory ball mill machine MM200..11

Section 1: TDA Data treatment

The temporal variance σ^2 of the dispersion profile from the TDA experiment in frontal mode was obtained by fitting the elution front with a Gaussian error function (*erf*) (d'Orlyé et al., 2008; Taylor, 1953) using equation (S1):

$$y = \frac{1}{2} + \frac{1}{2} erf \frac{(\mathrm{t} - \mathrm{t}_0)}{\sigma\sqrt{2}} \tag{S1}$$

The sample's diffusion coefficient D and hydrodynamic radius R_h were obtained from equation (S2) and equation (S3) respectively.

$$D = \frac{R_c^2 t_0}{24\sigma^2} \tag{S2}$$

$$R_h = \frac{k_B T}{6\pi\eta D} \tag{S3}$$

where R_c is the capillary radius (m), t_0 is the average elution time (s), T is the temperature (K) and η is the viscosity of the carrier liquid (Pa s). The two conditions of validity of TDA expressed by τ and Pe numbers and calculated using inequations (S4) and (S5) (Taylor, 1954; Chamieh and Cottet, 2014; Cottet et al., 2014) were fulfilled:

$$\tau = \frac{D t_0}{R_c^2} \geq 1.25 \tag{S4}$$

$$Pe = \frac{u R_c}{D} \geq 40 \tag{S5}$$

where τ is an dimensionless characteristic time, Pe is the Peclet number and u is the linear mobile phase velocity (m s^{-1}). Inequation (S4) is verified when the characteristic diffusion time of the solute in the capillary cross section is much lower than t_0.

Inequation (S5) is valid when the axial diffusion of the solute is negligible compared to Taylor dispersion.

In order to measure the viscosity of the carrier liquid (or background electrolyte, BGE), the capillary was filled with BGE. Then, a 0.1% (v:v) DMF solution in BGE was continuously injected at 100 mbar. The DMF was detected at 200 nm. The mean elution time of DMF (t_{DMF}) was measured, and the relative viscosity (η) was calculated by comparing it to the elution time with pure water (t_0) at the same temperature. The viscosity, being proportional to the elution time, is given by equation (S6) with about 3% precision (Bello et al., 1994):

$$\eta = \frac{t_{DMF}}{t_0}\eta_0 \qquad \text{(S6)}$$

where η_0 is the viscosity of water at the same temperature. In this work, the viscosity of tris buffer was found to be similar to the viscosity of water (8.92×10^{-4} Pa s).

Section 2: Tables and figures

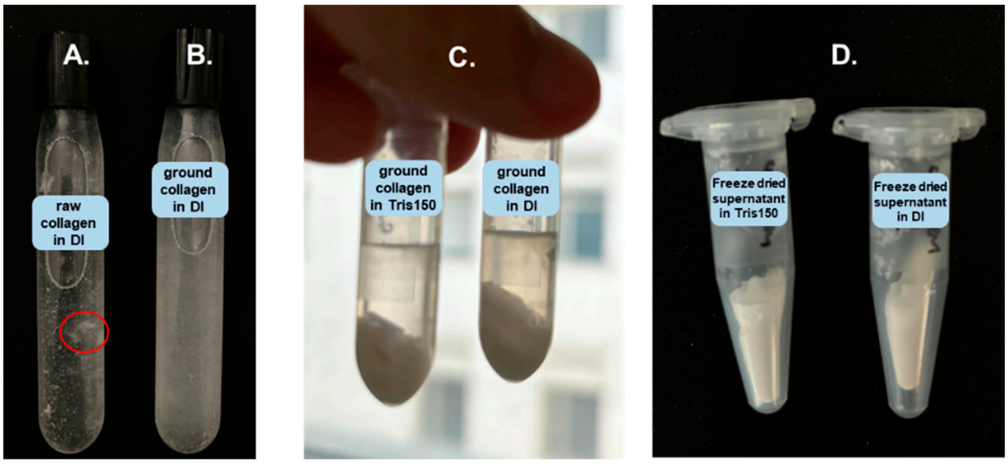

Figure S1. Photos of raw collagen 5 g/L in water (A), ground collagen 75 g/L in water (B) and ground collagen 75 g/L after centrifugation in 150 mM Tris-HCl buffer (C,

left) and in ultrapure water (C, right); Freeze dried supernatant collagen in 150 mM Tris-HCl buffer (D, left) and in water (D, right).

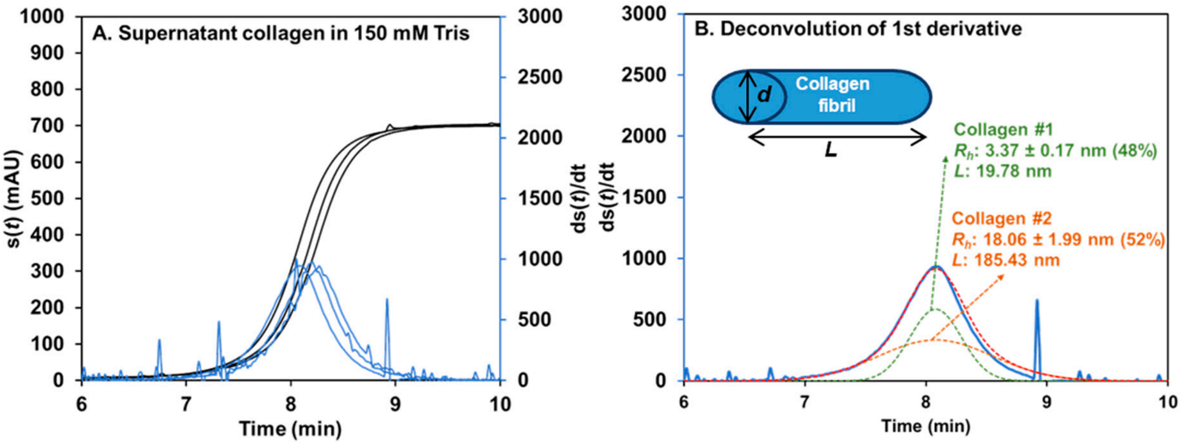

Figure S2. Frontal taylorgrams (black) and 1st derivative (blue) of supernatant collagen in 150 mM Tris with 36 mM NaCl pH 7.4 UV detection at 200 nm (A). Deconvolution of 1st derivative taylorgram with 2 gaussian curves (B). Gaussian fitting to calculate R_h is plotted as a red dotted line. Experimental conditions: fused silica capillary of 65 cm total length (56.5 cm to UV detector) × 50 µm i.d. Eluent: 150 mM Tris with 36 mM NaCl (pH 7.4). Mobilization pressure 100 mbar. Experiments were performed at 37°C.

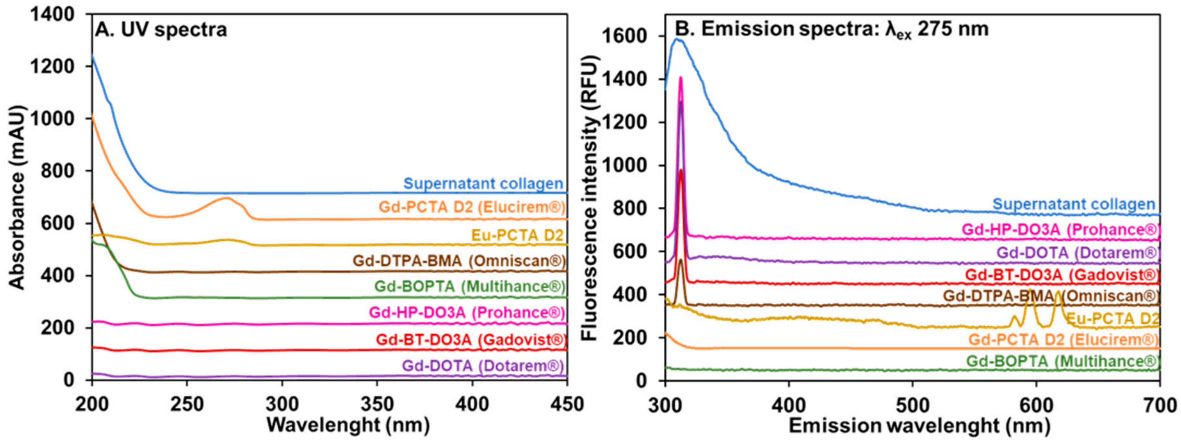

Figure S3. UV-Visible spectra (A) and emission spectra (B, λ_{ex} 275 nm) of 5 mM GBCA and supernatant collagen in 10 mM Tris-HCl buffer pH 7.4 except for Gd-DTPA-BMA and Gd-BOPTA diluted in ultrapure water. Sensitivity of spectrofluorometer was set at very low level. Cuvette pathlength was 1 cm.

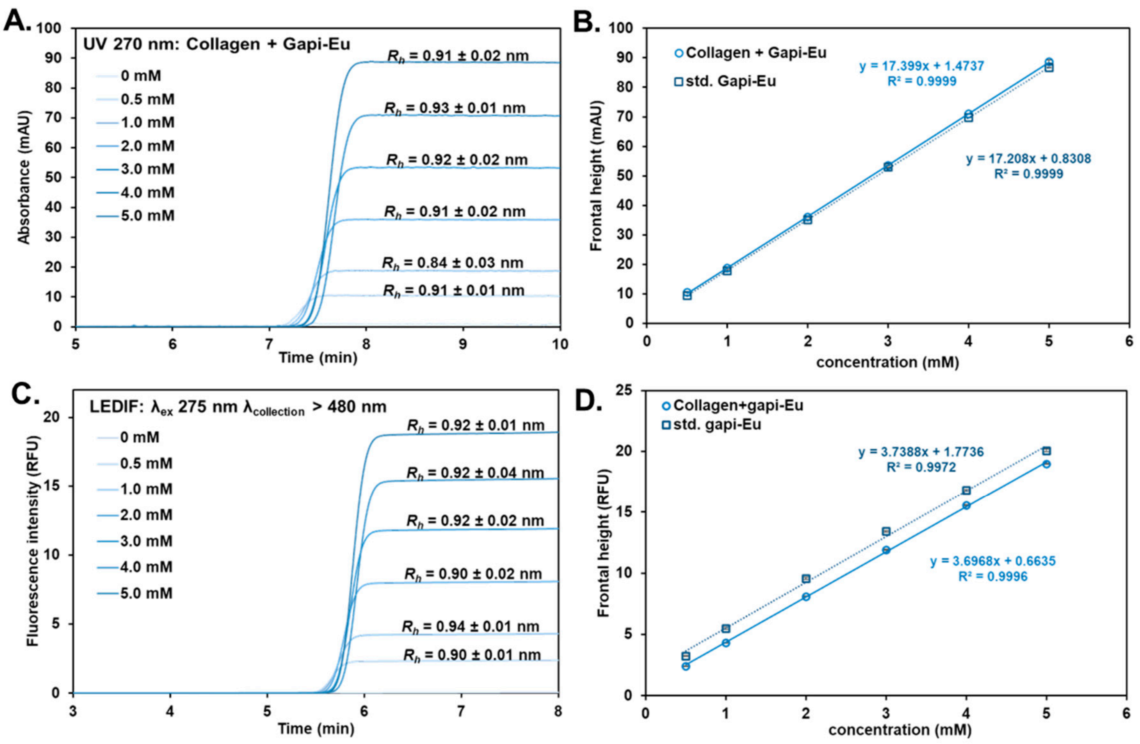

Figure S4. Frontal taylorgrams of Eu-PCTA D2 (0.5 – 5.0 mM) in the presence of ground collagen (75 g/L) and after centrifugation in UV mode at 270 nm (A) and using LEDIF detection (C). Linear calibrations of Eu-PCTA D2 obtained by TDA in frontal mode in the absence (dotted line) and in the presence (solid line) of ground collagen at UV 270 nm (B) and LEDIF detection (D). Experimental conditions: fused silica capillary of 65 cm total length (56.5 cm to UV detector) × 50 µm i.d. Eluent: 10 mM Tris-HCl buffer (pH 7.4). Mobilization pressure: 100 mbar. UV detection at 270 nm and LEDIF λ_{ex} 275 nm, $\lambda_{collection}$ >480 nm. Incubation of mixture: 37°C 1000 rpm for 4 h. Experiments were performed at 37°C.

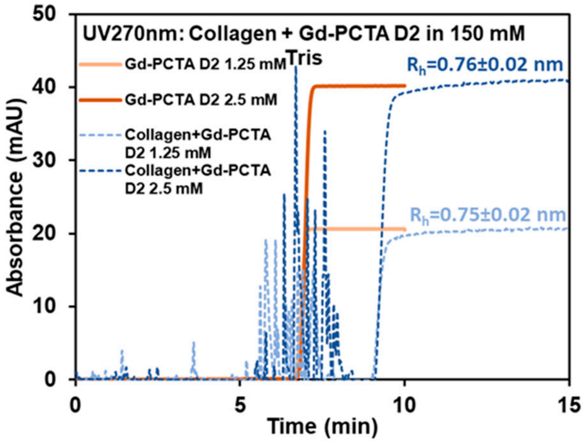

Figure S5. Frontal taylorgrams of Gd-PCTA D2 (1.25 and 2.5 mM) after interaction with ground collagen (75 g/L) and centrifugation (dotted lines). Standard of Gd-PCTA D2 (solid line). Experimental conditions: fused silica capillary of 65 cm total length (56.5 cm to UV detector) × 50 µm i.d. Eluent: 150 mM Tris buffer with 36 mM NaCl (pH 7.4). Mobilization pressure: 100 mbar. UV detection at 270 nm. Incubation of mixture: 37°C, 1000 rpm for 4 h. Experiments were performed at 37°C.

Table S1. Frontal taylorgrams of GBCA before (plain line) and after filtration (dotted line) with UV detection at 200 nm testing the retention of GBCA on the centrifugal filter (Amicon and Pall)**.** All GBCA were diluted in 10 mM Tris-HCl buffer pH 7.4 except for Gd-DTPA-BMA and Gd-BOPTA diluted in ultrapure water. Experimental conditions: fused silica capillary of 65 cm total length (56.5 cm to UV detector) × 50 µm i.d. Eluent: 10 mM Tris buffer (pH 7.4). Mobilization pressure: 100 mbar. UV detection at 200 nm. Incubation of mixture: 37°C 1000 rpm for 4 h. Sample volume: 60 µL. TDA experiments were performed at 37°C.

<table>
<tr><th rowspan="2">GBCA</th><th colspan="2">Retention on filter testing</th></tr>
<tr><th>Amicon 10 kDa
(2.5 mM GBCA)</th><th>Pall 10 kDa
(2.5 mM GBCA)</th></tr>
<tr><td>Gd-DTPA-BMA (Omniscan®)</td><td>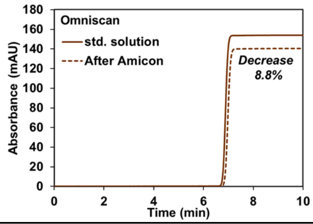
</td><td>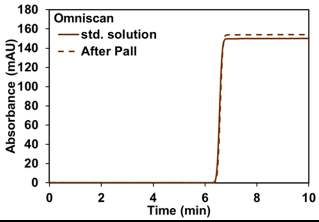
</td></tr>
<tr><td>Gd-HP-DO3A (Prohance®)</td><td>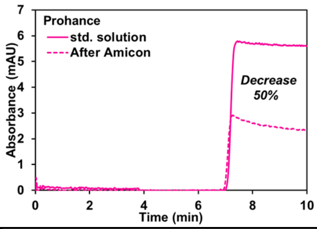
</td><td>N.I.</td></tr>
<tr><td>Gd-BT-DO3A (Gadovist®)</td><td>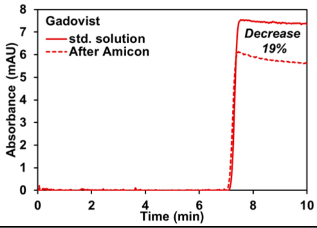
</td><td>N.I.</td></tr>
<tr><td>Gd-PCTA D2 (Elucirem®)</td><td>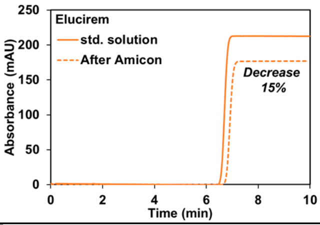
</td><td>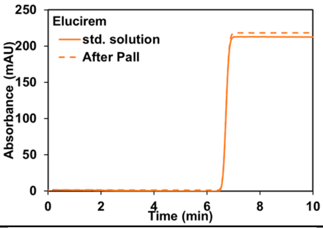
</td></tr>
<tr><td>Gd-DOTA (Dotarem®)</td><td>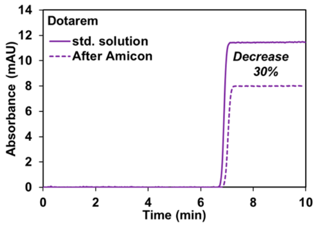
</td><td>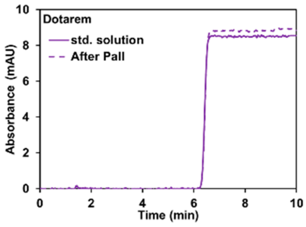
</td></tr>
</table>

Gd-BOPTA (Multihance®)	N.I.	N.I.

N.I.: Not investigated

Table S2. Frontal taylorgrams and its corresponding first-derivative obtained for GBCA alone (first column), supernatant collagen (second column), experimental mixture between GBCA and supernatant collagen at the same final concentration as individual (third column), and overlay of all the taylorgrams showing the absence of significant interactions. Experimental conditions: fused silica capillary of 65 cm total length (56.5 cm to UV detector) × 50 µm i.d. Eluent: 10 mM Tris buffer (pH 7.4). Mobilization pressure: 100 mbar. UV detection at 200 nm. Incubation of mixture: 37°C 1000 rpm for 4 h. Sample volume: 60 µL. TDA experiments were performed at 37°C.

<table>
<tr><th colspan="2">Individual</th><th>Experimental mixture</th><th>Sum of taylorgram</th></tr>
<tr>
<td>Gd-PCTA D2 (Elucirem®) 2.5 mM
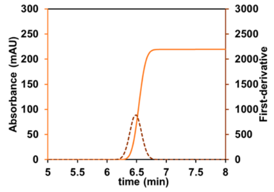

R_h = 0.73 nm</td>
<td>Supernatant collagen 1.22 g/L
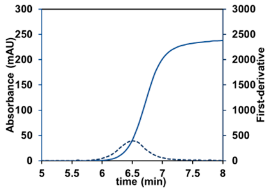

R_h = 4.95 nm</td>
<td>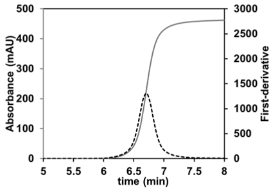

R_h = 0.72 nm (62%)
, 5.07 nm (38%)</td>
<td>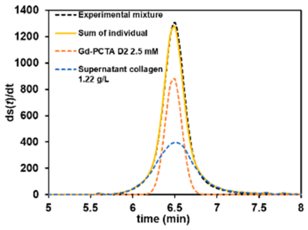
</td>
</tr>
<tr>
<td>Gd-DOTA (Dotarem®) 4.0 mM
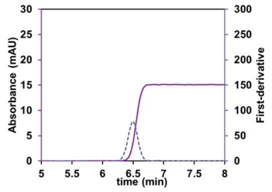

R_h = 0.44 nm</td>
<td>Supernatant collagen 0.084 g/L
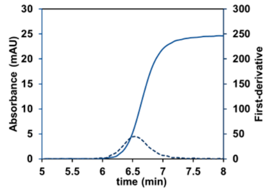

R_h = 4.01 nm</td>
<td>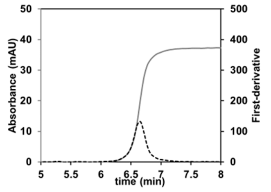

R_h = 0.43 nm (50%)
, 3.98 nm (50%)</td>
<td>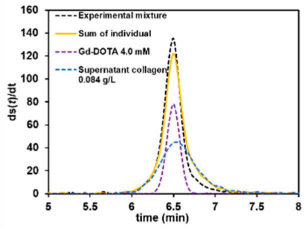
</td>
</tr>
<tr>
<td>Gd-BT-DO3A (Gadovist®) 4.0 mM
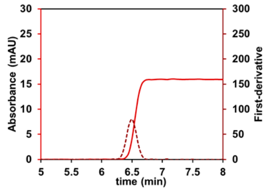

R_h = 0.45 nm</td>
<td>Supernatant collagen 0.09 g/L
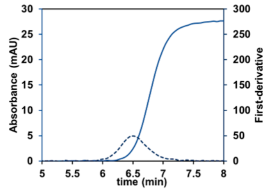

R_h = 3.99 nm</td>
<td>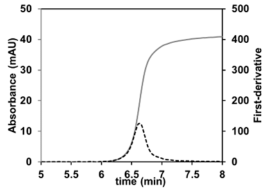

R_h = 0.52 nm (54%)
, 3.95 nm (46%)</td>
<td>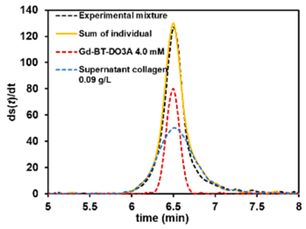
</td>
</tr>
</table>

Gd-HP-DO3A (Prohance®) 4.0 mM

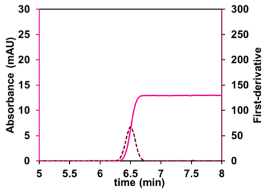

R_h = 0.43 nm

Supernatant collagen 0.08 g/L

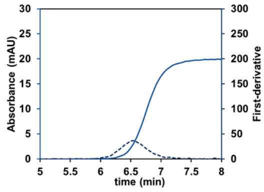

R_h = 3.96 nm

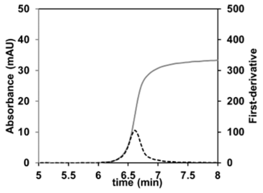

R_h = 0.47 nm (55%), 4.0 nm (45%)

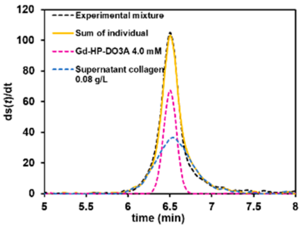

Gd-DTPA-BMA (Omniscan®) 2.5 mM

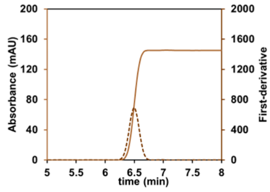

R_h = 0.51 nm

Supernatant collagen 0.81 g/L

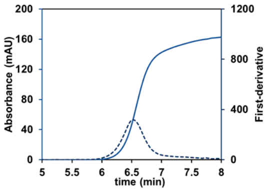

R_h = 3.49 nm

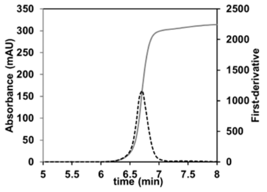

R_h = 0.58 nm (64%), 4.10 nm (36%)

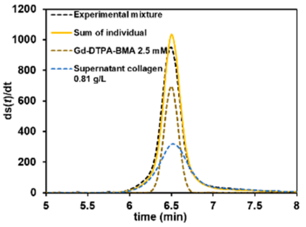

Gd-BOPTA (Multihance®) 2.5 mM

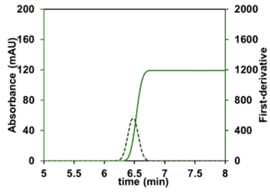

R_h = 0.51 nm

Supernatant collagen 0.66 g/L

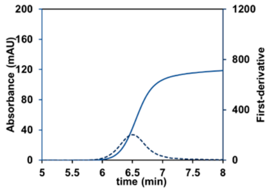

R_h = 4.75 nm

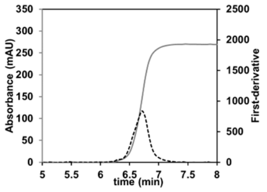

R_h = 0.55 nm (65%), 4.92 nm (35%)

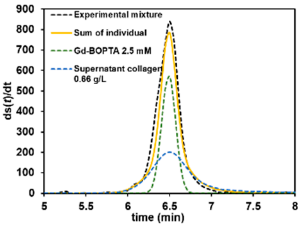

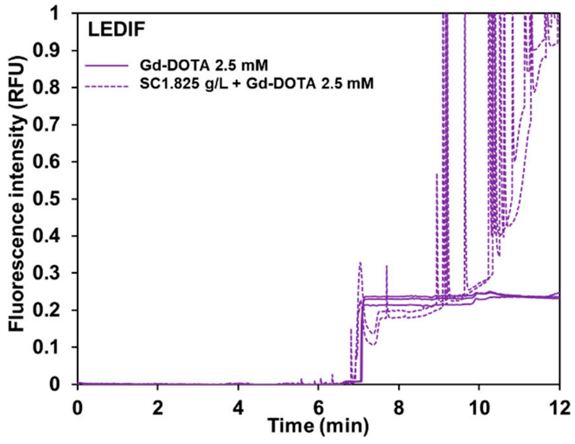

#

Figure S6. Time-scale frontal electropherograms obtained by LEDIF detection of 2.5 mM Gd-DOTA standard (plain lines) and their mixtures in the presence with 1.825 g/L supernatant collagen (dotted lines). Experimental conditions: PDADMAC coated capillary of 65 cm total length (56.5 cm to UV detector) × 50 µm i.d. Eluent: 150 mM Tris with 36 mM NaCl buffer (pH 7.4). LEDIF λ_{ex} 275 nm, $\lambda_{collection}$ 300-450 nm.

Incubation of mixture: 37°C 1000 rpm for 4 h. Applied voltage: -15 kV (from inlet) for standard GBCA and applied co-pressure +50 mbar (from inlet) for supernatant collagen in mixture with GBCA. Experiments were performed at 37°C. Concentrations indicated on the Figures are final concentrations after mixture.

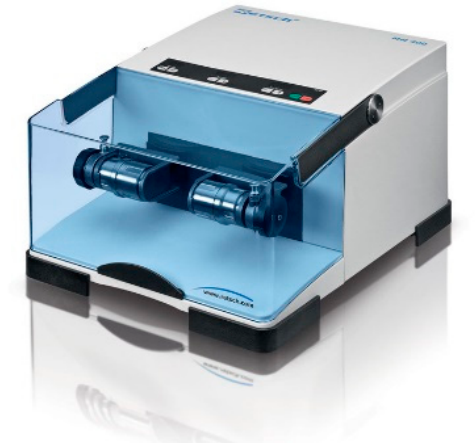

Figure S7. Vibratory ball mill machine MM200 (Retsch, France). Operating conditions: 4500 rpm for 4 min.
